# On the Comparative Value of Ergot of Rye and Galvanism in Obstetric Practice

**Published:** 1854-10

**Authors:** 


1. *On the comparative value of Ergot of Rye and Galvanism in obstetric practice.* By R. BARNES, M. D., Lecturer on Midwifery at the Royal Free Hospital Medical School. (*The Lancet*, 5th and 12th November, 1853.)

2. *On Galvanism as an obstetric agent.* By THOMAS RADFORD, M. D., Consulting Physician to the Manchester and Salford Lying-in Hospital. (*The Lancet*, 26th November, 1853.)

1. Dr. Barnes' principle object in the paper under consideration is to show the complete superiority of galvanism over ergot in the management of labor characterized by defective uterine action; and in doing this he brings to light much important matter.

In the first place he sets himself to show the dangers arising out of the unmanageable contraction which is set up by the ergot—rupture of the uterus and of the perinæum, laceration of the os uteri, prolapsus of the uterus and bladder, dangerous depression of the mother, murder to the child, and so on. Of these dangers, more or less serious, the two last are the least understood, and they are also best elucidated by Dr. Barnes.

The fact that ergot always causes depression, and often serious depression, is not generally understood, and yet, as we take it, this is the all-important fact in accounting for its action upon the uterus. According to our own views in the matter, the uterus should contract under the influence of ergot, not in consequence of any *stimulation* exercised by the drug upon the uterine fibres, but in consequence of a directly contrary influence. Hence the depression referred to is an all-important and significant fact in accounting for its action. Upon this point Dr. Barnes quotes from Dr. Hardy:—

"Dr. Hardy relates," he says (*Dublin Quarterly Journal*, 1845,) "that in several cases where the circulation had undergone depression from the action of ergot, the effect continued for several days, notwithstanding that in some instances inflammation of the uterus followed delivery, and the uterine tumor not unfrequently remained much larger than natural, even where there was no inflammation." Again; "Drs. Hardy and M'Clintock have observed a marked diminution of the mother's pulse in from fifteen to twenty minutes after the administration of the ergot; and all concur in noticing the dangerous depression following the use of ergot when given in cases where the powers of the system have been reduced by hemorrhage. In one such case ergot was almost immediately followed by most alarming symptoms, and depression requiring the most powerful stimulants. In several cases the depressed state of the circulation continued several days."

The injury done to the child offers a very tangible objection. While it continues, the contraction of the uterus necessarily suspends more or less the fœtal circulation, by interfering with the due aëration of the fœtal blood. This happens during a natural uterine pain, but the duration of this pain is such that, as a rule, no damage results to the child. It is different, however, if this pain is prolonged inordinately, as it is by ergot. Dr. Barnes puts the case very well:—

"Drs. Hardy and M'Clintock observed that the pulsations of the fœtal heart underwent a similar diminution in frequency to that witnessed in the mother, and that this was succeeded by irregularity and intermission, and that it became *inaudible.* Dr. Hardy, Dr. Beatty, and others, after careful observation directed to this point, assert that unless the child be born within a limited interval from the administration of the drug, it will be still-born. The excessive mortality of the children in ergotic labor is a fact well established, although disputed by some practitioners enthusiastic in the praises of ergot. The Prefect of the Seine had observed an almost regular annual increase in the number of still-born children, and he was informed that in a large number of these cases ergot of rye had been given during labor. He put the following question to the Academy of Medicine: "What may be the influence of ergot of rye on the lives of infants, and on the maternal life?" The report made by a commission of the Academy consisting of Orfila, Adelon, Villeneuve, Mérat, and Danyau, contained the following conclusion: "Ergot of rye administered improperly causes death to the fœtus and injury to the mother." The immediate source of danger to the fœtus is either the toxical property imparted to the blood, or the interruption to the circulation through the uterus and the placenta, occasioned by the long-continued contraction of the uterus. In this latter case the child may perish from asphyxia. These are the usual sources of danger; but there is a third. The long-continued and violent pressure to which the child is subjected during ergotic labor may compress the brain beyond the limit of endurance, or it may impede the circulation through the umbilical cord. The toxical agency of the ergot upon the fœtal heart is exemplified in the observations already referred to of Dr. Hardy. The influence of contraction of the womb in arresting the circulation through the placenta, and consequently the fœtal circulation, has been demonstrated to me by actual observation. The case is so interesting, and the opportunity of making a similar physiological experiment must be so rare, that I will cite it in detail.

"CASE 2.—A woman, with an extremely contracted pelvis, and who ten years before had been delivered by craniotomy by Dr. Waller, consulted me about her condition. She was again pregnant. I became satisfied of the propriety of inducing premature labor; and the agent I determined upon employing was galvanism. Having waited until it was estimated that seven months of gestation had passed, the operation was commenced. I shall have to relate presently the course of the labor under the use of galvanism, and may therefore pass at once to the particular point it is my present wish to illustrate. When labor had set in, and the os uteri was partially expanded, the cord came down into the vagina. The pains being of a languid, uncertain character, the galvanic stimulus was kept up. The pulsations of the cord were strong, and 80 in the minute. Galvanism was applied during the pains; the contractions were sensibly increased in force, and during the contractions the pulsations in the cord became intermitting, and occasionally stopped. As the pain went off, and as the galvanism was discontinued, the pulsations resumed their former strength and regularity. I then tried the effect of galvanism in the absence of a pain. Contractions were induced, and the intermittence of the pulse followed.

"I then observed the effect of a pain uninfluenced by galvanism. The intermittence of the pulse was the same. I repeated these observations several times, and always with the same result. Toward the termination of the labor a strong expulsive pain came on, during which the head, which was very small, was driven into the vagina, without, however, causing any pressure upon the cord. During the strong pain the pulsation in the cord stopped entirely, but returned when the pain went off.

"But fœtal circulation is arrested during the physiological contraction of the womb for a short time only, and is completely restored during intervals sufficiently long to insure the safety of the child. In ergotic contraction the interruption is total, unremitting, and protracted. Shall we wonder if the child occasionally perishes from asphyxia?

"Dr. Ramsbotham, whose experience in the use of ergot in inducing premature labor is probably greater than that of any other practitioner, says—"After a great number of trials, I observed that although the mothers recovered as well as if through an ordinary labor, their systems not being in any sensible degree injuriously affected by the drug, yet that the proportion of children born still was greater than when the membranes were punctured. This I attributed to the baneful influence of the medicine upon the fœtus." Dr. Ramsbotham modified his practice in consequence. He further says that "Wright's experiments prove decisively that the medicine has a most prejudicial influence upon the young *in utero*, even to their destruction.

"If the child survives the perils of ergotic labor, is it free from subsequent danger?

"Dr. Ramsbotham says—"It has happened to me in four different instances to witness the death of the fœtus, a few hours after birth, by convulsions, after the induction of premature labor by ergot."

In the second place, Dr. Barnes pleads in favor of galvanism as a substitute for ergot, his chief reason being that the contraction which it provokes is perfectly manageable. In illustration of the efficacy of galvanism for this purpose, he refers to the evidence already published, particularly that by Dr. Radford and Mr. Haighton; and in addition to this he adduces some evidence derived from his own experience or from that of his friends.

Of the use of galvanism in inducing premature labor he mentions two cases:—

"Case 3.—I have already referred to this case for the purpose of illustrating the effect of contraction of the uterus upon the fœtal circulation. The result, although perfectly satisfactory, was by no means so speedily accomplished as in the case of Horninger and Jacobi. I had previously endeavored to bring on labor by puncturing the membranes, and inserting a sponge-plug in the cervix uteri. This proceeding was followed by no symptom of labor. On the 23d of January I applied the galvanic battery for half an hour, placing one pole on either side of the uterus. Immediately after commencing the shocks the bladder was irresistibly emptied, to the evident annoyance of the patient. The womb was felt to become hard, and the patient herself was sensible of contractions and increased movements of the fœtus. The contractions did not continue on the cessation of the galvanism, and I therefore repeated the application on the 24th and 26th, for about an hour each time. On the 26th a 'show' took place. On the evening of the 27th slight pains were felt; the cord was presenting, a small loop coming through the os uteri, which was now dilated to the size of a shilling, but feeling rigid. She had had rather copius flooding in the day-time, but it had stopped. The head was felt laying on the pubic in front of the os uteri, the cord coming down in the free space behind it. On this morning of the 28th, the galvanism having been applied at intervals all night, the pains had increased. I have already mentioned how the galvanism increased or originated contraction. At nine A. M. the child was born. It was apparently not more than six months old. The patient had certainly reckoned falsely. The child's heart was pulsating; the chest made three or four convulsive heaves, at which the mouth opened, but no air seemed to enter; the lungs refuse to expand; the walls of the chest were drawn in toward the spine. I endeavoured to excite respiration by the galvanic apparatus, but although I could at will cause a respiratory *effort*, the child was evidently too immature to live. The womb contracted favorably, and the placenta being withdrawn was found healthy. The patient recovered without a bad symptom.

"The excellent effect of galvanism in this case led me to recommend the use of the same agent to my friend, Mr. Mansford, who has favored me with the following account:—

"CASE 4.—'The lady, whose case led me to attempt the induction of premature labor, was in the forty-first year of her age, and the thirteenth week of her fifth pregnancy. On the 8th of November, 1852, having ruptured the membranes, I introduced one wire of the apparatus within the os uteri, and placed the other in contact with the spine. From the one introduced into the uterus I had removed the brass handle, and twisted the wire upon itself so as to form a loop sufficiently curved to insure its remaining steadily in its proper place. I also carefully enveloped a considerable portion of this wire with lint, as well to protect the vagina from the twisted portion and extremity as to prevent the galvanic current from being diverted from the uterus. I then increased its power until it produced "the most severe cutting pains in the loins," "great bearing down," and "a dreadful commotion in the womb." These were my patient's own expressions. The operation was repeated on the 9th and 10th, each morning for half an hour; the effect, however, had not been as yet altogether satisfactory, as I had not been able to maintain a continuous action; but on the fourth morning—viz., the 11th—I remedied this defect, and kept up a continuous current for three quarters of an hour, when my patient begged me to desist, which I did, and determined to wait a few days to see if this might accomplish the desired effect. Happily on the 14th, without any further interference, labor commenced, and terminated within four hours in the birth of a living child, and not a single untoward symptom occcured, spontaneously. It wasaltogether a most satisfactory case."

Of the use of galvanism in inertia during the first and second stages of labor, a case of Dr. Mackenzie is referred to:—

"CASE 5.—'I was sent for one morning to a young woman who had been admitted in labor at the Paddington Infirmary, and on examination I found that the head presented. Although she had been several hours in labor, the os uteri was but little dilated. I saw her in the course of the same afternoon, but still found vary little dilatation. At ten P. M. but little progress had been made. I now determined to try the effect of galvanism, and applied one pole of a single current machine to the spine, and the other, by means of Radford's director, to the neck of the uterus. The current was from time to time intermitted, and uterine action of a vigorous character was excited. In about an hour a fine living child was born. So vigorous were the expulsive efforts during the passage of the head through the os externum, that I was obliged to take particular pains to prevent rupture of the perinæum. The impression left on my mind by this case was that galvanism should not be employed except very cautiously in primiparæ, or in any other instance in which the perinæum is rigid or imperfectly developed."

In illustration of the use of galvanism in the third stage of labor, and in hemorrhage, another case by Dr. Mackenzie is referred to:—

"CASE 6.—'The patient had been upward of forty-eight hours in labor, under the care of Dr. Keogh, who had called in Mr. Clark, by whom I was sent for. When I saw the patient, uterine action had entirely ceased, and I found, on examination, that the head was impacted in the pelvis, the face presenting with the chin to the left cotyloid cavity. As the patient was exhausted, an opiate had been given, and as she was disposed to sleep, we agreed to meet again in some hours, and if uterine action did not return, to deliver by the forceps. At the appointed time no return of uterine action had taken place. I applied the forceps; the operation was accomplished with extreme difficulty, and the woman was delivered of a fine, large, living child. I left the patient shortly afterward, but the next day, on meeting Dr. Keogh and Mr. Clark, I learned that great apprehension had been felt throughout the night as to the occurrence of hemorrhage, inasmuch as the uterus had remained flaccid and uncontracted, and at the time of my visit it reached above the umbilicus, and was very soft and flabby. I proposed galvanism, and applied one pole to the spine and the other to the neck of the uterus, occasionally intermitting the current. This was done for half an hour, and evident uterine action was excited, the uterus becoming harder and smaller, and on removing the poles two large coagula were expelled. The next day the uterus was more contracted and smaller, and no hemorrhage had occurred. Galvanism was again used for half an hour. The uterus certainly contracted under its influence. The following day no hemorrhage had occurred, and the condition of the uterus was such as not to require any further recourse to the agent. The woman from this time recovered in a most favorable manner."

Dr. Barnes, also, relates a case in which he used galvanism with success for the purpose of expelling hydatids.

"CASE 7.—Ann W——, æt. 42, had eight children and three abortions. She applied to Mr. Forbes, on the 17th of June last, having anasarca of the legs. Two months before, she suffered a burning pain in the region of the womb. She had menstruated up to Christmas last. Since that date there had been a little hemorrhagic discharge at intervals. For the last month there has been a continual discharge of colored fluid. Her health is much impaired, and her strength lowered. On the 18th, while in bed, she felt a vaginal discharge, and on getting up passed a large quantity of blood. The pulse was weak, thready, 108; face blanched; headache intense. No pain preceded the hemorrhage. There was a tumor in the seat of the pregnant womb, extending more to the right side, and reaching to the umbilicus; it was firm and elastic, tender on pressure, which did not bring on labor-pains. The os uteri was the size of a shilling, and rigid. No placental murmur or sound of fœtal heart were heard. The breasts were quite flaccid. Os slightly expanded toward the afternoon. A dead fœtus, or some diseased condition of the ovum, was suspected. In consultation, Dr.

Barnes suggested galvanism to cause contraction; this had the desired effect, and Mr. Forbes was enabled to bring down a bunch of hydatids. The vagina was then plugged, and the abdomen bandaged. The disposition to contraction thus given, more hydatids were afterward passed. Tincture of ergot of rye was then given in small doses. Early on the morning of the 19th, the patient passed a large mass of hydatids, which was expelled suddenly with a pain like that of labor. She was quite exhausted with loss of blood and previous disease; symptoms of inflammation appeared, and she sank the same night. The *post-mortem* examination revealed a large fibrous tumor in the walls of the uterus, and an advanced stage of granular degeneration of the kidney."

The paper ends with a description of the mode in which galvanism is to be applied, and a summary of its advantages in comparison with ergot of rye:—

"The ordinary electro-magnetic apparatus in use for medical purposes is, I believe, the best form that can be employed. The principle of this apparatus consists in the induction of magnetic currents by a current of electricity, and the production of a rapid succession of feeble shocks by continual interruptions to the current. I have observed that the uterine contractions are always provoked at the break and renewal of the circuit. Repeated shocks act as a far more effectual and certain stimulus to uterine contractility than a continued current. It is probably through inattention to this fact that some practitioners have failed in effecting contraction of the uterus by means of galvanism. As to the mode of applying the poles, I do not think it necessary to apply one over the spine, and the other to the neck of the uterus, as is usually done. I have found the application of the disks, covered with thin flannel moistened in water, one on either side of the abdomen over the uterus, much more convenient and quite as effectual. The practice of applying one pole over the spine and the other to the neck of the uterus further seems to me to be based upon an erroneous view of the mode in which galvanism acts upon muscular fibre. When the poles are thus applied, one to the spine and the other to the cervix uteri, it is doubtful whether the ensuing contraction of the uterus is due to primary excitation of the spinal marrow."

"Among the advantages of galvanism more especially worthy of attention are:—

"1st. The simplicity of the operation.

"2d. The extensive range of cases in which it may be successfully employed, rendering the electro-magnetic apparatus a desirable addition to the armamentarium of the obstetric practitioner.

"3d. The perfectly manageable character of the agent. Its action may be broken off and renewed at pleasure. The moment we think the uterus is acting too powerfully under its use, we may instantly withdraw the exciting agency, and leave the uterus to the ordinary physiological stimuli, which seldom impel the organ to undue activity. It moreover admits of easy regulation; both the strength and duration of this agent are completely under our command. We have it in our power to imitate in a remarkable manner the natural pains, both as to intensity and intermission. Ergot has neither measure nor certainty.

"4th. Its peculiar appropriateness and efficacy in cases of extreme exhaustion of the system, where deglutition is difficult or impossible, or where the stomach rejects everything; where any other mechanical application to the uterus is dangerous or inconvenient, and especially where the introduction of the hand into the uterus would be likely to be attended by injury or even a fatal result. Indeed, it may be truly said that in cases of extreme exhaustion galvanism is the last resource left to us. The galvanic stimulus can be applied when everything besides is out of the question. The uterine muscular fibre will respond to this stimulus when the nervous system is utterly prostrate, when the heart has ceased to beat, when the patient is moribund or even dead.

"5th. Galvanism is less exhausting to the system than ergot or most other means of exciting contraction. It acts directly upon the uterine muscular fibre, and scarcely taxes at all the general powers of the system.

"6th. It does not necessarily preclude or supersede the use of other remedies tending to fulfill the same indication."

2. Dr. Radford's communication owes its origin to Dr. Barnes'. Its prime object is to vindicate the writer's claim to having been the first to recommend and employ galvanism as an obstetric agent in this country, as well as to state the kind of cases in which this agent has been employed by him. These cases are:—

1st. In cases of tedious labor arising from uterine inertia.

2d. In cases of accidental hemorrhage, either before or after the rupture of the membranes, and especially when exhaustion from loss of blood exists.

3d. In cases of "placenta prævia," in which the practice of detaching the placenta is adopted, and the vital powers are greatly depressed.

4th. In case of internal flooding before or during labor.

5th. In case of *post-partum* floodings.

6th. In cases of hour-glass or irregular contraction of the uterus.

7th. To originate, *de novo*, uterine action, or in cases in which it is desired to induced premature labor.

8th. In cases of abortion, when the indications show the necessity or justify the expulsion of the ovum.

9th. In cases of asphyxia in infants.

